# Supplementary figures and images for: Renal cancer cells acquire immune surface protein through trogocytosis and horizontal gene transfer
Source: PLoS One. 2025 May 29;20(5):e0325043. doi: 10.1371/journal.pone.0325043 (PMC12121793; doi:10.1371/journal.pone.0325043)

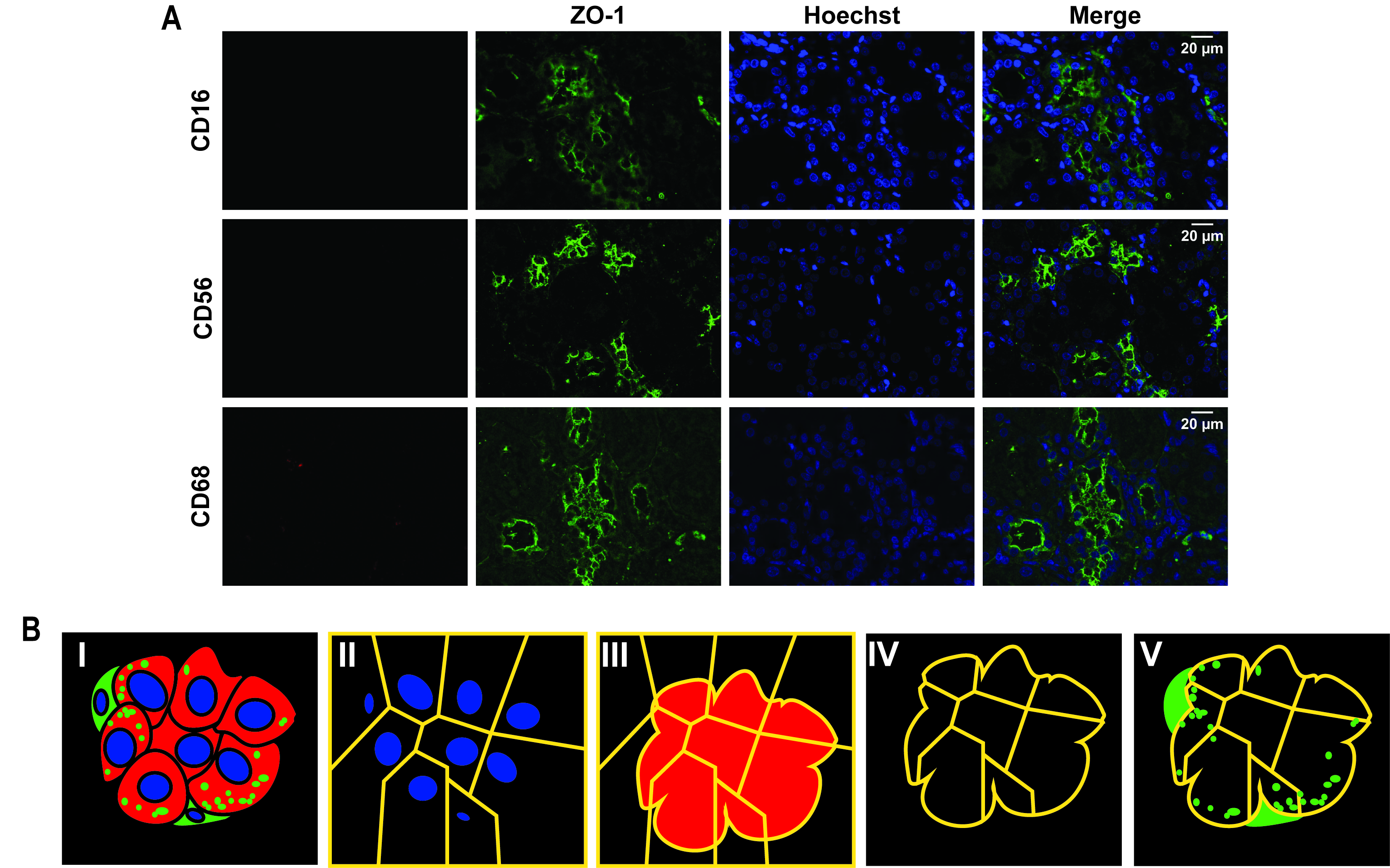

Supplement: S1 Fig — Trogocytic markers shown in the left panel, ZO-1 staining highlights normal kidney epithelial cells. (B) Representative illustration of the algorithm used to quantify immunofluorescent kidney slides. (B,I) Cancer cells represented in red (CAIX), lymphocytes represented in green (CD45). Trogocytic tumor cells represented by expression of both CAIX and CD45. (B,II) Detection of nuclei through hoechst staining, Voronoi diagram is applied based on the location of nuclei and segments cells. (B,III and IV) CAIX staining is applied to the previous voronoi diagram and used to exclude non-CAIX+ cells such as lymphocytes and non-tumor tissue. (B,V) Final voronoi diagram is applied to CD45 labeling to approximate the boundaries of cells and determine the expression levels of CD45 on tumor cells only. (TIF) [file pone.0325043.s001.tif]

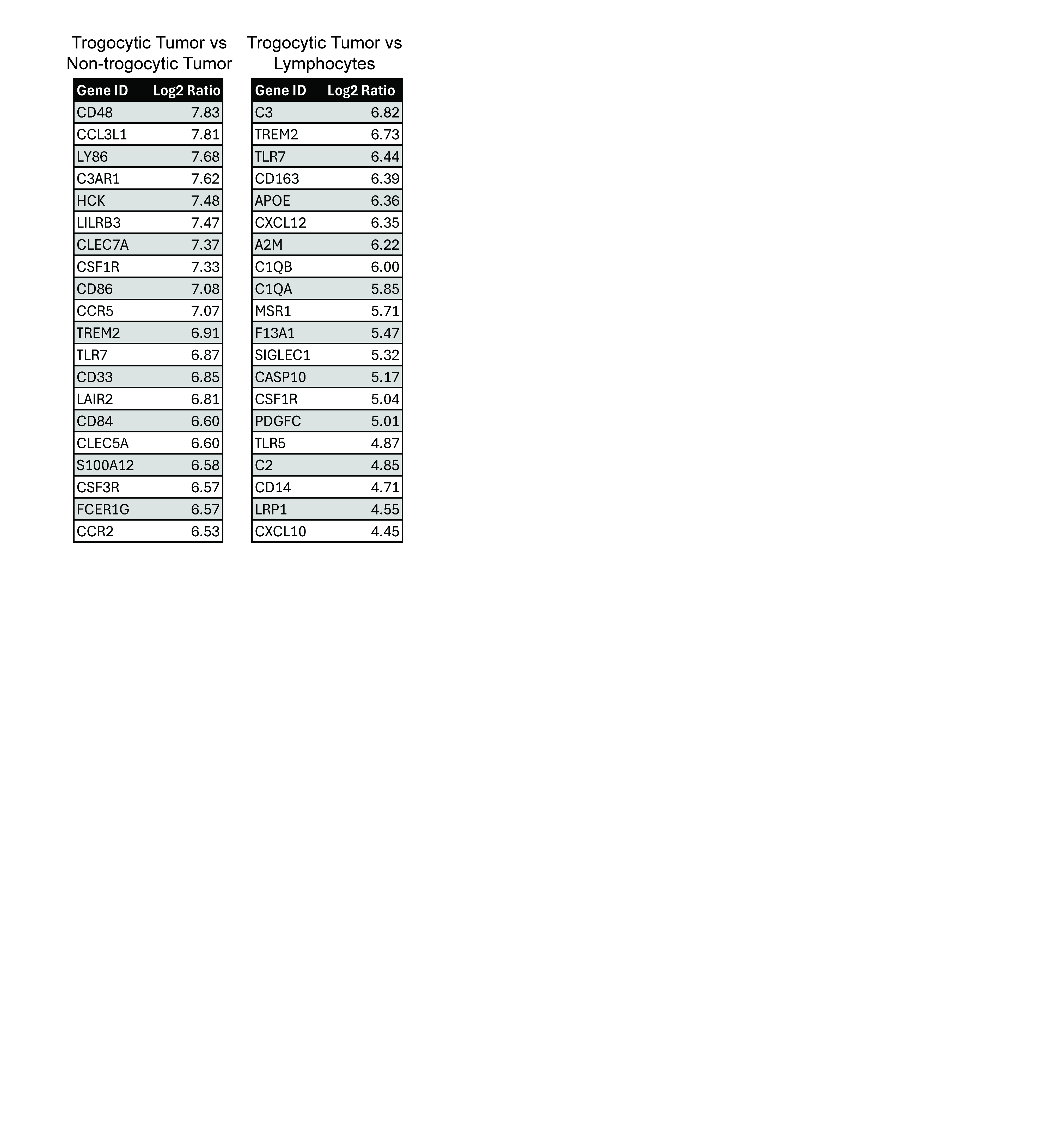

Supplement: S1 Table — (Left) Results show a comparison of the most differentially upregulated genes in trogocytic tumor cells relative to non-trogocytic tumor cells based on the PanCancer Immune Profiling Nanostring® panel. (Right) Top 20 differentially upregulated genes in trogocytic tumor cells relative to tumor infiltrating lymphocytes. Results are based on mean gene expression counts of 3 non-trogocytic tumor cell populations, 4 trogocytic tumor cell populations, and 5 tumor infiltrating lymphocyte populations isolated from human ccRCC tumors. (TIF) [file pone.0325043.s002.tif]

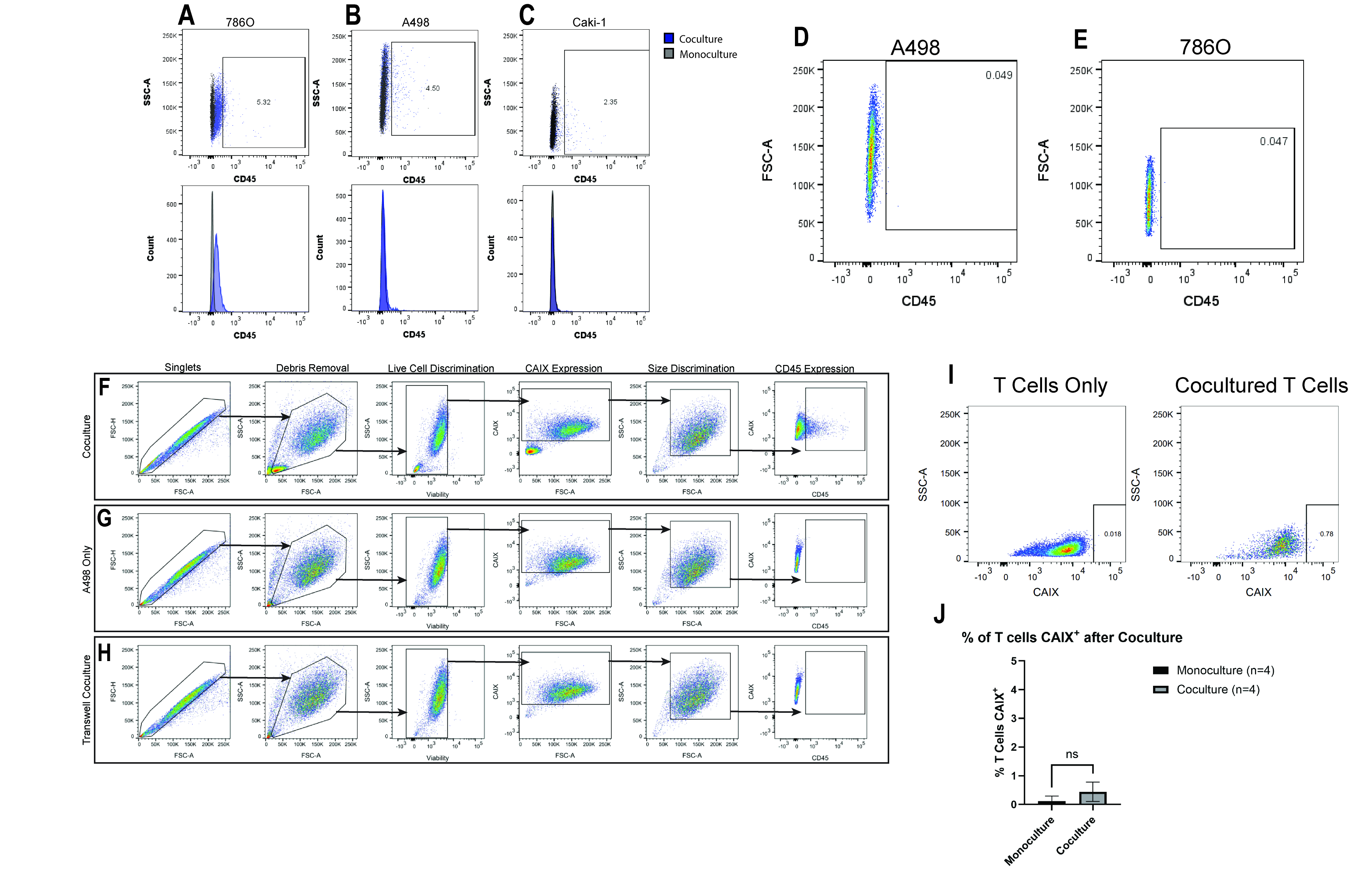

Supplement: S2 Fig — (Top) Gating indicates the percentage of RCC cells that are CD45+ relative to monoculture controls. (D,E) Flow cytometry analysis of A498 and 786O cocultures with primary human T cells that were separated by a transwell barrier. Gating indicated the percentage of RCC cells that were positive for CD45 post-over night coculture. (F-H) Flow cytometry gating strategy used to remove T cell for analysis of CD45 positive cancer cells. Example depicts A498 cells cocultured with primary T cells (F), A498 cells only (G), and A498 cells separated from primary T cells using a transwell barrier (H). (I) Representative plot of CAIX transfer from tumor cells to T cells post coculture with Caki-1 cells. (J) Quantitative comparison of %CAIX+ T cells post coculture with Caki-1 cells showing no significant transfer of CAIX (T-test, p value = 0.135). (TIF) [file pone.0325043.s003.tif]

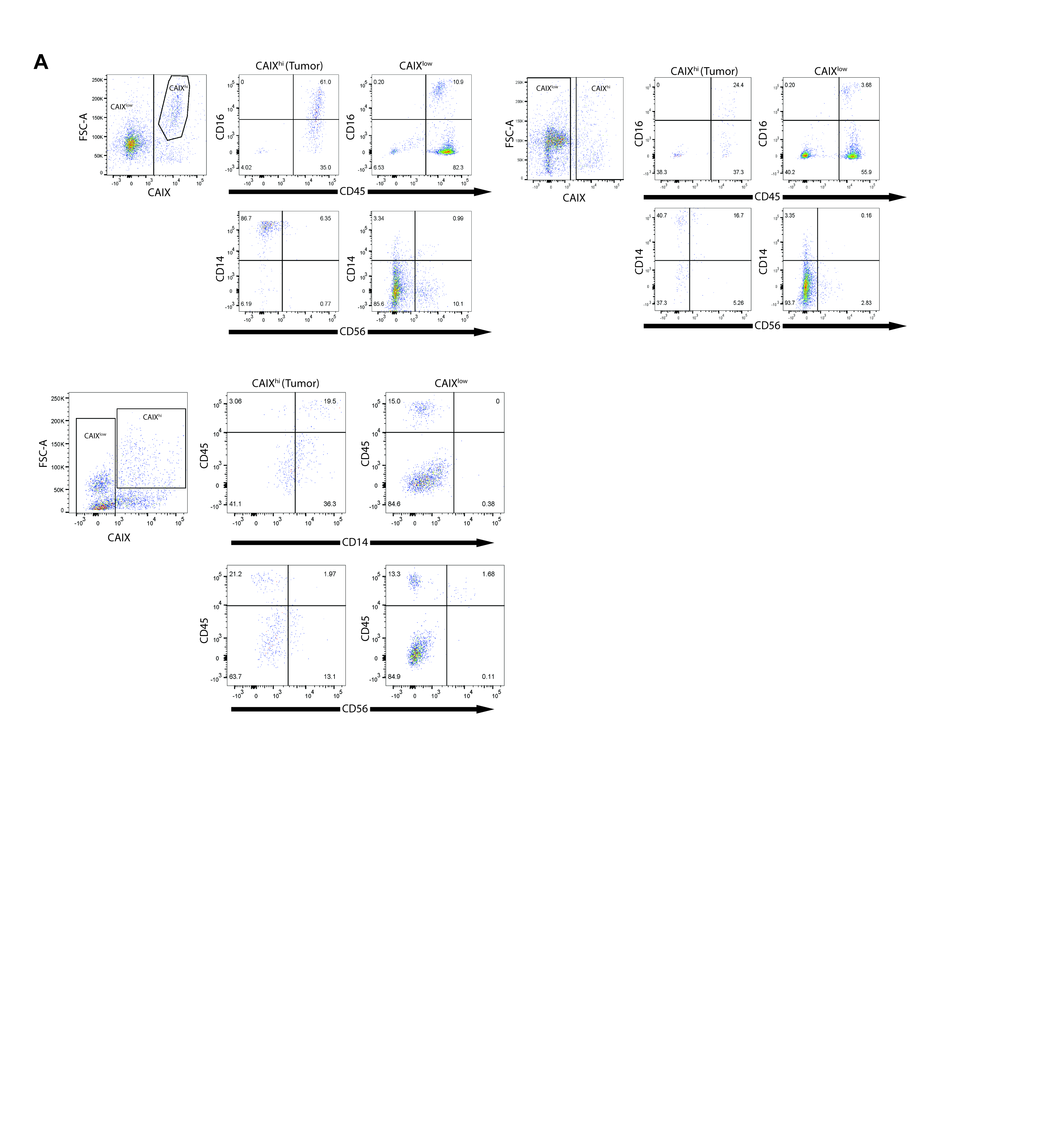

Supplement: S3 Fig — Left plots indicate the gating strategy used to isolate CAIX+ tumor cells and CAIXlow lymphocytes. The right plots for each tumor indicate the percentage of cells in these two populations that are positive for the indicated immune cell proteins. (TIF) [file pone.0325043.s004.tif]

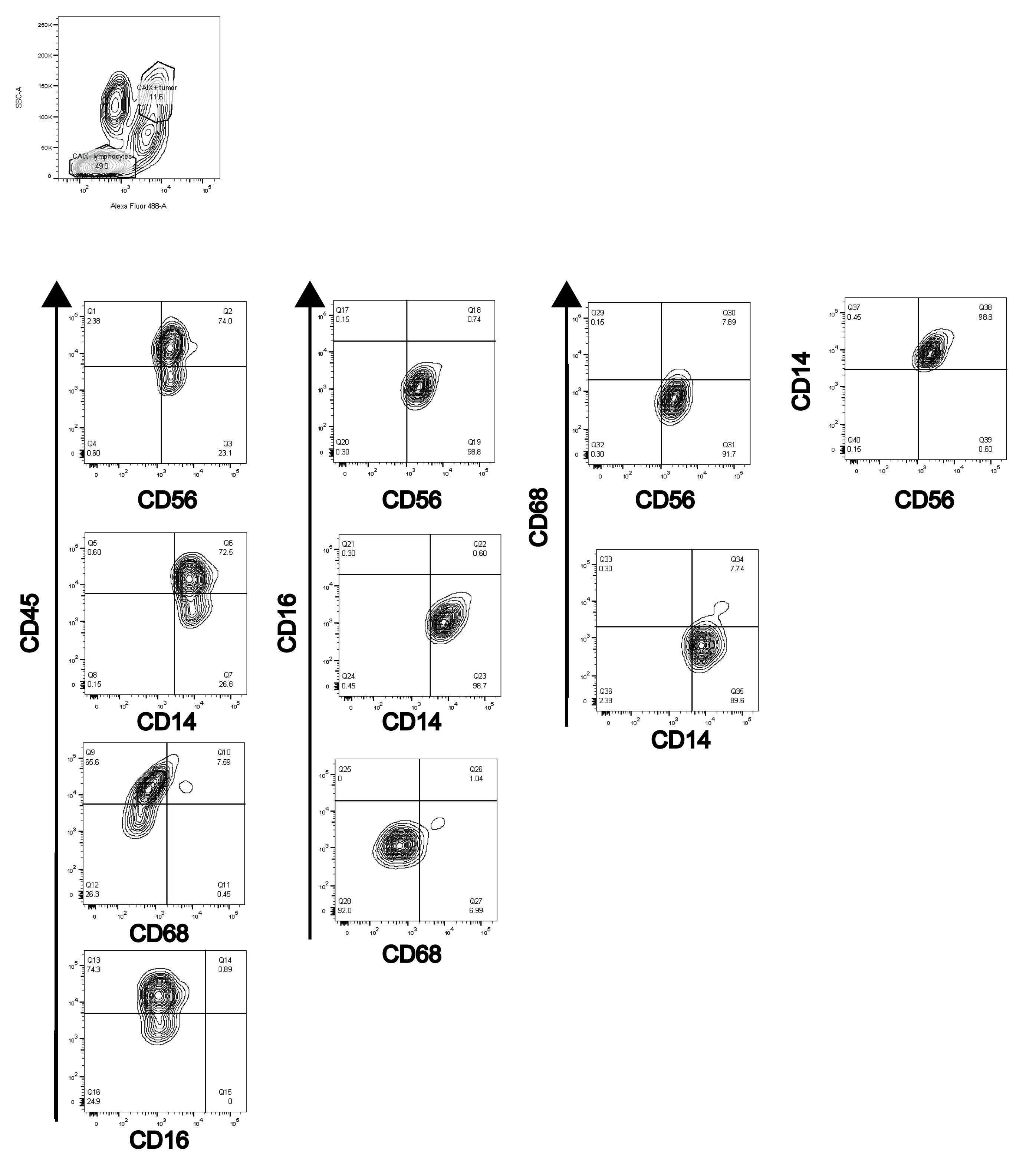

Supplement: S5 Fig — Prior to analysis, tumor cells were isolated based on CAIX+ expression, and size discrimination. (TIF) [file pone.0325043.s006.tif]

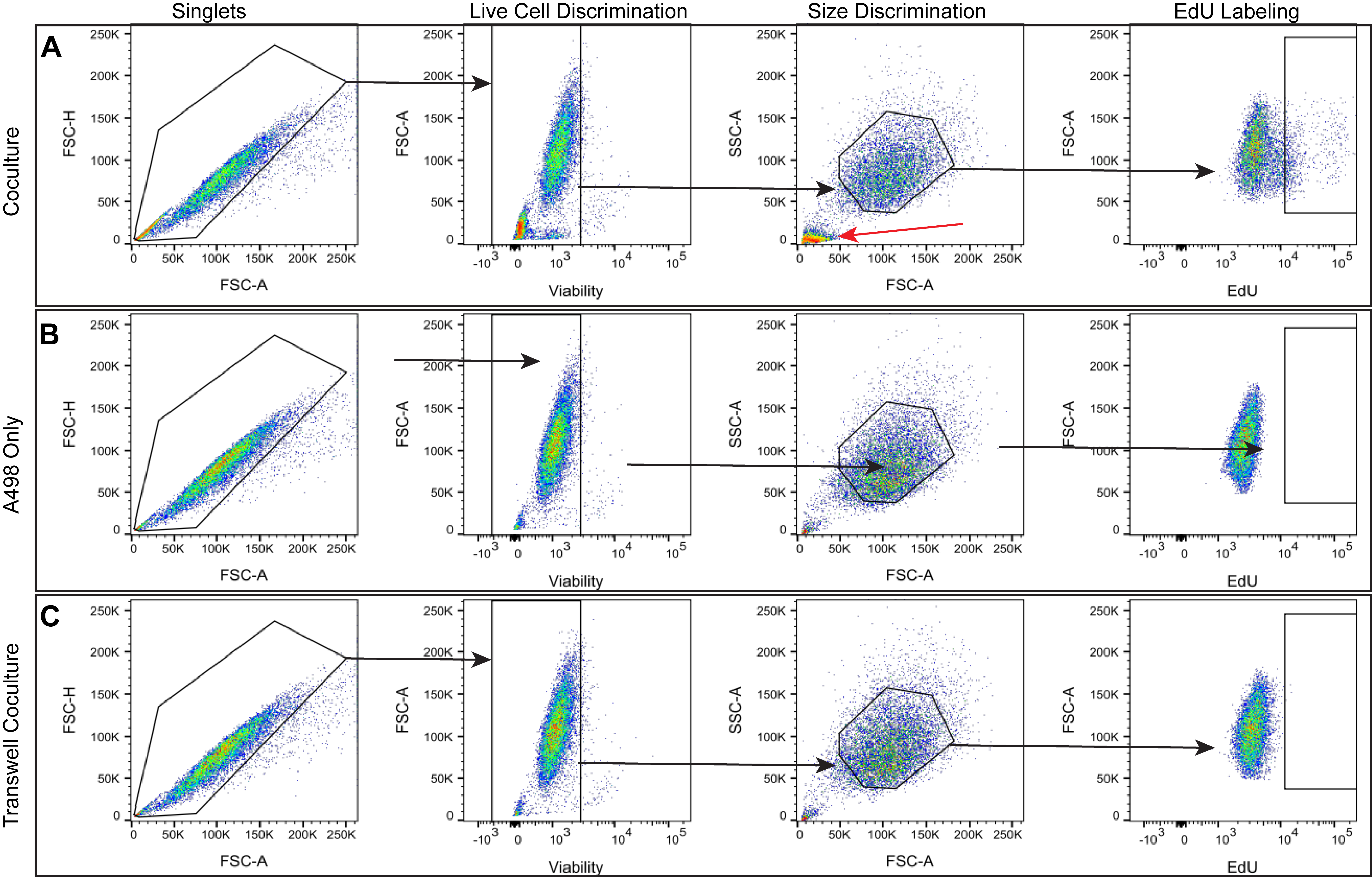

Supplement: S6 Fig — (A) Single cells in coculture suspension were identified using FSC-H FSC-A discrimination. Live cells were determined using UV Ghost Dye 450 as a marker for viability. SSC-A and FSC-A were used for identifying T cells and RCC cells, subsequently isolating RCC cells only for analysis. Red arrow indicates the position of T cells. FSC-A and EdU plot depicts the gate used to identify EdU positive cancer cells. (B) Gating strategy applied to a monoculture of only A498 cells. (C) Gating strategy applied to an A498-T cell coculture that was separated by a transwell barrier. T cells were removed with the barrier prior to flow cytometry analysis. (TIF) [file pone.0325043.s007.tif]
