# Supplementary material for: Renal cancer cells acquire immune surface protein through trogocytosis and horizontal gene transfer
Source: PLoS One. 2025 May 29;20(5):e0325043. doi: 10.1371/journal.pone.0325043 (PMC12121793; doi:10.1371/journal.pone.0325043)
Supplement: Attachment — Submitted filename: PONE-D-24-53647_comments.docx [file pone.0325043.s008.docx]

Marcarian et al. discovered that human clear cell renal carcinoma can gain immune cell markers through both trogocytosis and horizontal gene transfer. Although trogocytosis was reported in several previous research, most of them focus on antigen transfer from tumor cells to immune cells. This finding provides a novel perspective in the field of cancer immunology and tumor microenvironment. However, the molecular mechanism of trogocytosis and how tumor benefit from gaining immune cell markers need further studied. I believe after moderate revision experiments and polishing to the text and figures, this could be a good fit for PLOS ONE.

Major concerns:

1. Trogocytosis discovered by previous studies were mediated by some level of interaction of the surface molecules between the two cells (TCR to MHC-peptide complex; CAR-antigen; CTLA4-B7 complex). What interacting molecules are responsible for trogocytosis of immune cells markers to tumor cells? Based on the result, trogocytosis discovered by the authors are stochastic and random.
2. To what extend this type of trogocytosis rely on the nature/characteristic of immune cells? Would you still observe trogocytosis if you coculture two tumor lines, with one of them overexpress immune cell marker like CD45?
3. Previous studies have shown that trogocytosis happens in both directions. Is this type of trogocytosis discovered by the authors also reciprocal (meaning that tumor markers like CA9 also found transferred to immune cells?

Minor concerns:

1. Figures should be citated/callout sequentially. However, Figure 3C was called out before Figure 3A and 3B.
2. Figure 2D:
   a. N = 4 in the legend but there are only three data points for CD16. Please explain where the missing data point goes.
   b. This figure is not very good at demonstrating the claim made in the result section: “*Our data revealed that the vast majority of CAIXhi cells were positive for multiple immune cell markers.*” It’s better to use one or multiple bar plots or pie charts to show the percent of quadrupole, triple, or double positive cells for each sample. For example, 30% CD45 positive and 30% CD14 positive doesn’t necessary mean 30% of the cells are double positive for these two markers. Very likely these 30% were not the same group of cells.
   c. It would be better if use different symbol for different samples (or connected same sample with line). With this additional information, it would be interesting to see whether some sample have higher trogocytosis for all markers consistently.
3. Figure 3A only shows gating strategy for Lymphocytes, Non_trogocytic tumor, and Trogocytic tumor. It can be moved to supplemental.
4. Figure 3B Nanostring data would be better to include more immune markers. For example, Figure 3C and figure 3D both show upregulation of makers like CD45, CD14, and CD16.
5. The authors should cite more current research articles in the introduction part. Recommended high impact paper to cite:
   a. Huang, J. F. et al. TCR-Mediated internalization of peptide-MHC complexes acquired by T cells. Science 286, 952–954 (1999).
   b. Hamieh, M. et al. CAR T cell trogocytosis and cooperative killing regulate tumour antigen escape. Nature 568, 112–116 (2019).
   c. Zhou, X., Cao, H., Fang, SY. *et al.* CTLA-4 tail fusion enhances CAR-T antitumor immunity. *Nat Immunol* **24**, 1499–1510 (2023).
   d. Schriek, P. et al. Marginal zone B cells acquire dendritic cell functions by trogocytosis. *Science* **375**, eabf7470 (2022).
